# Evaluation of *in vitro* cytotoxic effect of *Trichosanthes dioica* root

**DOI:** 10.4103/0974-8490.75454

**Published:** 2010

**Authors:** Sanjib Bhattacharya, Pallab K. Haldar

**Affiliations:** *Bengal School of Technology (A College of Pharmacy), Sugandha, Hooghly 712102, West Bengal, India*; 1 *Himalayan Pharmacy Institute, Majhitar, Rangpo, East Sikkim 737136, India*

**Keywords:** *Allium cepa*, antimitotic, cytotoxic, root

## Abstract

**Background:**

*Trichosanthes dioica* Roxb. (Cucurbitaceae), called pointed gourd in English is a dioecious climber grown in India and used traditionally for various medicinal purposes.

**Methods:**

Present study was aimed to evaluate *in vitro* cytotoxic effect of dichloromethane (DCTD), methanol (METD), and aqueous (AQTD) extracts of *T. dioica* root using *Allium cepa* root meristems by keeping them in different concentrations of each test extract under specific experimental conditions followed by determination of root growth inhibition (root length and number) and mitotic index.

**Results:**

All the extracts significantly demonstrated concentration-dependent inhibition of root length and number and reduction in mitotic index, indicating antimitotic activity demonstrating cytotoxicity and genotoxicity. DCTD was found to be the most potent (EC_50_ : 2.8 mg/ml), followed by METD and AQTD.

**Conclusion:**

The present study therefore, establishes promising *in vitro* cytotoxic and genotoxic property of *T. dioica* root against the test system.

## INTRODUCTION

*Trichosanthes dioica* Roxb. (Cucurbitaceae), called pointed gourd in English, *Potol in* Bengali, *Palval* in Hindi, and *Patola* in Sanskrit, is a dioecious climber found wild throughout the plains of north and North-East India from Punjab to Assam and Tripura states. It is particularly cultivated in Uttar Pradesh, Bihar, West Bengal, and Assam states of India, for its fruits, a common culinary vegetable in India. In India, all parts of this plant have been used traditionally for several medicinal purposes. According to Ayurveda, the traditional system of Indian medicine, its root is a purgative. The root has traditionally been used as a hydrogouge cathartic, tonic, and febrifuge, and in the treatment of jaundice, anasarca, and ascites.[1–4] However, there are no reports on the pharmacological studies on its root. In our earlier studies, we have reported on the nematocidal effect of the leaf and root of *T. dioica*.[5–6] Present study attempts to evaluate *T. dioica* root for its *in vitro* cytotoxic effect using *Allium cepa* root meristems to explore the promise of *T. dioica* for possible antitumor efficacy.

## MATERIALS AND METHODS

### Plant material

The mature tuberous roots of *T. dioica* were collected during December 2008 from Majdia, Nadia district, West Bengal, India. The species was identified by Dr. M. S. Mondal at the Central National Herbarium, Botanical Survey of India, Howrah, West Bengal, India, and a voucher specimen (SB-02) was deposited at the Pharmacognosy Research Laboratory, Bengal School of Technology (A College of Pharmacy), West Bengal, India. Just after collection, the plant material was washed thoroughly with water and shade dried at room temperature (24–26°C), and ground mechanically into a coarse powder.

### Chemicals

All the chemicals used were of analytical grade, obtained from Merck.

### Preparation of extracts

The powdered plant material (750 g) was initially macerated with *n*-hexane (1 L) overnight, and the air-dried marc was macerated separately with dichloromethane (DCM), methanol (MeOH), and distilled water (450 mL each) at room temperature (24–26°C), with frequent shaking for 4 days, followed by re-maceration with the solvents, similarly for 3 days. The macerates were combined, filtered and evaporated to dryness *in vacuo* (at 35°C and 0.8 Mpa) to yield DCM (3.72%), MeOH (7.22%), and aqueous extracts (11.05%), which were denoted as DCTD, METD and AQTD respectively. All the dry extracts were kept in a vacuum desiccator until use. Preliminary phytochemical analysis revealed the presence of flavonoids, triterpenoids and steroids in DCTD. METD revealed the presence of flavonoids, triterpenoids, steroids, saponins, amino acids, carbohydrates and reducing sugars, whereas AQTD indicated the presence of flavonoids, saponins, carbohydrates, reducing sugars and amino acids.[7]

### Test samples

Test samples for *in vitro* cytotoxic bioassay were prepared freshly from the dry extracts. Varying concentrations of all the test extracts, viz. DCTD (1, 2, 4, 8, 16 mg/ml), METD (20, 40, 80, 160, 320 mg/ml), and AQTD (100, 200, 400, 800, 1600 mg/ml) were prepared by dissolving or suspending in double-distilled water and sonicated for 10 min immediately before use.

### Evaluation of *in vitro* cytotoxicity (Allium test)

This study was conducted as per the methods reported by previous workers with requisite modifications.[8,9]

### *Allium cepa* bulbs

Approximately equal size bulbs (40 ± 10 g) of the onions (*Allium cepa* L.) were obtained from the local Market at Chendernagore, West Bengal. Any onions that were dry, moldy or have started shooting green leaves were discarded.

### Growing *Allium cepa* meristems

The outer scales were removed from the healthy onion bulbs leaving the root primordia intact. These bulbs were grown in dark for 48 h over 100 ml of tap water at ambient temperature until the roots have grown to approximately 3 cm. The water was changed daily during this period. The viable bulbs were then selected and used for subsequent studies.

### Exposure to test samples

The bulbs with root tips grown up to 2-3 cm were removed from the water and placed on a layer of tissue paper to remove excess of water. The bulbs were divided into 18 groups (*n* =12), each six groups for each test extract. The first group for each extract served as control. Immediately, the bulbs were placed in test tubes filled with test samples (one bulb in each) and control (tap water) and incubated at 22 ± 2°C for 96 h away from direct sunlight. The test samples were changed daily with fresh ones. The length of roots grown during incubation (newly appearing roots not included), root number and the mitotic index were recorded after 96 h. After this period the root appearance was also noted. The effective concentration for 50% root length inhibition (EC_50_) was determined by plotting treatment concentration against root length as percentage of control.

### Microscopic studies and determination of mitotic index

After 96 h, the root tips (2-3 mm) were collected and prepared for microscopic studies by standard aceto-orcin squash preparation technique.[10] For each root tip, the numbers of mitotic cells and total meristematic cells were counted in 5-8 fields of view using high resolution (100×) bright field light microscopy. Mitotic index was represented in terms of dividing cells/total cells and expressed as percentage.

### Statistical analysis

The data are presented as the mean ± Standard Error of Mean (SEM). The data were analyzed for statistical significance by Student’s ‘*t*’ test. *P* values less than 0.05 (*p* ≤ 0.05) were considered as statistically significant.

## RESULTS

The results for cytotoxic effects of different extracts from *T. dioica* root are summarized in Tables 1–3. All the test extracts exhibited significant (*P* < 0.001) inhibitory effects on root growth (root length and number) and reduction in mitotic index (antimitotic effect) in a concentration dependent manner. DCTD was the most potent, producing inhibitory action at minimum concentrations employed (1-16 mg/ml) on root length and number and significant decrease in mitotic index. Its EC_50_ value was found to be minimum i.e. 2.8 mg/ml. Similarly, METD was moderately active at comparatively higher concentrations (20-320 mg/ml). Its EC_50_ value was 122 mg/ml. AQTD was the least active only at higher concentration ranges (100-1600 mg/ml). It exhibited similar inhibitory responses at concentrations of 800 and 1600 mg/ml without significant changes in mitotic index. Its EC_50_ value could not be determined. The test samples did not cause any change in color of roots, however at 4 and 8 mg/ml concentrations of DCTD the color of roots turned pale brownish and became blackish brown at 16 mg/ml of DCTD. The morphology of root tips was not affected at any concentrations of any test extract.

**Table 1:** Influence of DCTD on root length, root number and mitotic index of *Allium cepa* roots

Concentration (mg/ml)	Mean root ength ± SEM	Mean root number ± SEM	Mitotic index (%) ± SEM
Control (tap water)	31.7 ± 0.13	31.1 ± 0.23	64.6 ± 0.41
1	21.8 ± 0.27¶	30.7 ± 0.52	54.3 ± 0.19¶
2	17.3 ± 0.30*	24.6 ± 0.18¶	41.2 ± 0.27*
4	13.6 ± 0.38*	18.2 ± 0.45*	28.5 ± 0.36*
8	9.6 ± 0.49*	13.1 ± 0.20*	16.2 ± 0.51*
16	6.7 ± 0.62*	11.2 ± 0.37*	4.2 ± 0.45*

Data are expressed as mean ± SEM (n = 12);

¶ *P* < 0.05 and

* *P* < 0.001 compared to control.

**Table 2:** Influence of METD on root length, root number and mitotic index of *Allium cepa* roots

Concentration (mg/ml)	Mean root ength ± SEM	Mean root number ± SEM	Mitotic index (%) ± SEM
Control (tap water)	30.4 ± 0.09	34.01 ± 0.22	63.7 ± 0.29
20	27.5 ± 0.25¶	27.2 ± 0.13¶	57.2 ± 0.30¶
40	22.2 ± 0.16*	25.1 ± 0.32*	44.6 ± 0.24*
80	18.7 ± 0.20*	21.2 ± 0.48*	40.2 ± 0.15*
160	12.2 ± 0.34*	15.1 ± 0.28*	25.7 ± 0.55*
320	11.6 ± 0.23*	14.8 ± 0.30*	24.2 ± 0.39*

Data are expressed as mean ± SEM (n = 12);

¶ *P* < 0.05 and

* *P* < 0.001 compared to control.

**Table 3:** Influence of AQTD on root length, root number and mitotic index of *Allium cepa* roots

Concentration (mg/ml)	Mean root ength ± SEM	Mean root number ± SEM	Mitotic index (%) ± SEM
Control (tap water)	30.7 ± 0.41	32.1 ± 0.44	62.9 ± 0.46
100	30.2 ± 0.17	29.9 ± 0.34	61.1 ± 0.37
200	24.5 ± 0.33¶	27.2 ± 0.11¶	50.2 ± 0.09¶
400	16.8 ± 0.22*	21.2 ± 0.26*	42.3 ± 0.32*
800	16.2 ± 0.25*	20.3 ± 0.42*	41.4 ± 0.18*
1600	16.2 ± 0.20*	20.1 ± 0.10*	41.1 ± 0.31*

Data are expressed as mean ± SEM (n = 12);

¶ *P* < 0.05 and

* *P* < 0.001 compared to control.

## DISCUSSION

In the present investigation, the *in vitro* cytotoxic effects of defatted DCM, MeOH and aqueous extracts of *T. dioica* were evaluated by using *Allium cepa* root meristem model (Allium test), where root growth inhibition and antimitotic effects provided the indication of cytotoxicty. Allium test is a rapid, highly sensitive and reproducible bioassay for detecting cytotoxicity and genotoxicity; and shows good agreement with results obtained from other test systems.[9,11] Here, cytotoxicity of all test extracts were evidenced by evaluating macroscopic parameters, i.e., reduction in root number and the most importantly, root length, both of which were indicative of root growth inhibition. The cytotoxic effect was further confirmed by microscopic studies involving determination of mitotic indices reduction of which allowed assessment of impaired cell division (antimitotic effect) thereby providing definitive information regarding the extent of cytotoxic action. Both growth inhibition and antimitotic effects were found to be in concert, as growth inhibition had been the inevitable consequence of diminished cell division.

DCTD exhibited maximum and remarkable cytotoxic potential followed by METD and AQTD. The EC_50_ values (calculated on the basis of root length inhibition) of DCTD and METD were 2.8 and 122 mg/ml respectively, while that of AQTD was not found. Thus AQTD could be potentially less cytotoxic while still inhibiting cell divisions at higher concentrations. There were irreversible changes in root tip cells resulting in their growth retardation and cessation. The change in color of DCTD treated roots to brownish was due to cytotoxic effects causing cell death.[11]

Preliminary phytochemical analysis revealed the presence of various compounds in METD, whereas the DCTD mainly contained triterpenoids and steroids. It appears that the presence of triterpenoids and/or steroids was responsible for the enhanced activity of DCTD. In this connection, it is noteworthy to mention here that METD also contained triterpenoids and steroids, along with several other constituents, but the expected synergistic effect, however, was not observed here, as DCTD was more active than METD. AQTD exhibited the weakest effect, possibly because of the absence of triterpenoids and steroids. Some synergy was obvious, indicating METD to be more active than AQTD, because of the presence of triterpenoids and steroids in METD. Nevertheless, activity in AQTD indicated that not only triterpenoids and steroids were responsible for the cytotoxic activity. Dereplication strategies based on these findings could be helpful for isolation of the active cytotoxic constituents.

With respect to these results it is apparent that *T. dioica* root contains cytotoxic constituents that can stop cell division. These constituents plausibly affected the cytoskeleton or inhibited the activity of one or more components of the cell cycle.

The present study confirms the *in vitro* cytotoxic effect of *T. dioica* root extracts in Allium test. The results indicate that the METD and DCTD especially, possess potential cytotoxic activity including marked antimitotic effect, but more positive results are definitely needed in other test systems involving *in vitro* and *in vivo* animal and human cancer cell lines for qualifying prospective anticancer activity. The present preliminary investigation provides comprehensive *in vitro* evidence that *T. dioica* root demonstrates remarkable cytotoxic and genotoxic properties thus suggesting the feasibility of its possible use as natural antitumor agent. Further antitumor studies on *T. dioica* root using animal cancer call lines are presently underway.
